# N-Acetyl-Seryl-Aspartyl-Lysyl-Proline Mitigates Experimental Colitis Through Inhibition of Intestinal Mucosal Inflammatory Responses *via* MEK-ERK Signaling

**DOI:** 10.3389/fphar.2020.00593

**Published:** 2020-05-06

**Authors:** Yingying Shi, Mingxia Zhou, Junkai Yan, Zizhen Gong, Jin Wu, Yuanwen Chen, Yingwei Chen

**Affiliations:** ^1^Department of Gastroenterology, Xinhua Hospital, School of Medicine, Shanghai Jiao Tong University, Shanghai, China; ^2^Department of Gastroenterology and Nutrition, Shanghai Institute of Pediatric Research, Shanghai, China

**Keywords:** N-acetyl-seryl-aspartyl-lysyl-proline, prolyl endopeptidase, colitis, intestinal epithelial cell, MEK-ERK signaling

## Abstract

N-acetyl-seryl-aspartyl-lysyl-proline (AcSDKP) is an endogenous immunomodulatory peptide that is generated from thymosin β4 (Tβ4) through stepwise hydrolysis, involving meprin-α and prolyl endopeptidase (PREP). It is well acknowledged that AcSDKP exerts beneficial effects on multiple cardiovascular and renal diseases. However, the functional role of AcSDKP in inflammatory bowel disease (IBD) remains poorly understood. Here, we aimed to assess the content of AcSDKP in patients with IBD and investigate the impact of AcSDKP on intestinal inflammation in IBD. We found that in the inflamed mucosal specimens of patients with ulcerative colitis, the expression levels of Tβ4 and meprin-α were decreased, while PREP was expressed at similar levels to non-inflamed mucosa. *In vitro*, AcSDKP inhibited the expression of proinflammatory factors in intestinal epithelial cells partially by reducing the activation of MEK-ERK signaling. *In vivo* studies showed that transgenic mice, with lower levels of AcSDKP, were more vulnerable to dextran sulfate sodium (DSS)-induced colitis and exhibited more severe intestinal inflammatory responses. On the other hand, exogenous AcSDKP infusion significantly attenuated the clinical symptoms and intestinal mucosal inflammation in DSS-induced mice. In conclusion, results from this study demonstrated the anti-inflammatory function of AcSDKP within the intestine and suggest that AcSDKP has a promising therapeutic potential for IBD treatment.

## Introduction

Inflammatory bowel disease (IBD) defines a group of complex disorders, characterized by chronic relapsing inflammation of the gastrointestinal tract, of which ulcerative colitis (UC) and Crohn's disease (CD) are the most typical forms. Since 1990, IBD has become a public health problem with increasing incidence in newly industrialized countries and rising prevalence in western countries (Ng et al., 2018). Although the specific pathogenesis of IBD remains unclear, it is well established that the chaotic patterns of cytokine networks, along with the aberrant trafficking of immune cells, play a central role in the inflammatory process of IBD (Friedrich et al., 2019; Neurath, 2019). In the past few decades, several biologic agents have been successfully introduced for the treatment of IBD refractory to conventional medications, such as the anti-TNF agents and anti-integrin agents (Neurath, 2017). However, there are still some limitations with respect to the successful use of these biologic agents in clinical practice. These include the high cost that imposes heavy economic burden on the patients, loss of effectiveness over time, and increased risk of opportunistic infections in the long-term (Danese et al., 2015). Therefore, it is imperative to search for safer and more efficient agents for IBD therapy, with clearly understood molecular mechanisms.

N-acetyl-seryl-aspartyl-lysyl-proline (AcSDKP) is a naturally occurring tetrapeptide that is ubiquitously distributed in the plasma, urine, and various organs. Originally reported as a hematopoiesis regulator that inhibits the proliferation of hematopoietic pluripotent stem cells (Frindel and Monpezat, 1989), AcSDKP is now implicated in many physiological processes, such as inflammation, fibrosis, angiogenesis, and apoptosis. Recently, AcSDKP has drawn considerable attention due to its anti-inflammatory properties (Kumar and Yin, 2018). Numerous studies have shown that AcSDKP exerts strong protective effects on several diseases involving the brain, heart, liver, and kidney; in part by reducing local inflammatory reactions (Chen et al., 2010; Worou et al., 2015; Zhang et al., 2017; Sharma et al., 2018). Additionally, taking into consideration that AcSDKP is mainly degraded by the N-domain of angiotensin-converting enzyme (ACE) (Azizi et al., 1996; Eriguchi et al., 2018), several studies reported that the anti-inflammatory and anti-fibrotic effects of ACE inhibitors on the heart and kidney, were at least partly mediated by increasing AcSDKP in plasma and tissues (Peng et al., 2007; Chan et al., 2018). Mechanistically, AcSDKP works through modulating multiple aspects of immune responses, including the activation of critical signaling pathways, release of proinflammatory mediators, infiltration of T-cells, and differentiation or migration of macrophages (Sharma et al., 2008; Gonzalez et al., 2014; Zhu et al., 2016; Li et al., 2017). These pleiotropic immunomodulatory functions of AcSDKP encouraged us to explore the potential role of AcSDKP in regulating intestinal inflammation, particularly in the context of IBD.

AcSDKP is derived from its precursor thymosin β4 (Tβ4) by the combined action of meprin-α and prolyl endopeptidase (PREP). Meprin-α is a zinc metalloproteinase that was deemed anti-inflammatory in the intestine, as meprin-α deficiency rendered mice to have more severe colitis (Banerjee et al., 2009). Recent studies reported that basal AcSDKP concentrations were significantly lower in meprin-α knockout mice compared to wild-type (WT) mice (Kumar et al., 2016). PREP is a serine protease that prefers to cleave small peptides, containing less than 30 amino acids (Gass and Khosla, 2007). Previous studies have reported that PREP inhibitor effectively prevented the release of AcSDKP from Tβ4 *in vitro* (Myohanen et al., 2011). Moreover, studies have demonstrated that PREP inhibitors decreased the endogenous levels of AcSDKP in the plasma and other tissues, including the heart and kidney, in rats (Cavasin et al., 2007). Hence, in our study, PREP knockout (PREP-KO) mice were generated to investigate whether a decreased level of AcSDKP is associated with the severity of intestinal inflammation in IBD models. Furthermore, we explored the effects of AcSDKP on the occurrence and development of experimental colitis by supplying exogenous AcSDKP. In addition, we also studied the roles and relevant mechanisms of AcSDKP in the inflammatory responses of intestinal epithelial cells (IECs).

## Materials and Methods

### Cell Culture and Treatment

Caco-2, a human colonic adenoma cell line, purchased from the Cell Bank of the Chinese Academy of Sciences (Shanghai, China), was incubated in DMEM, supplemented with 10% fetal bovine serum (Gibco, USA), 1% non-essential amino acids (Gibco, USA), and 1% penicillin/streptomycin. Caco-2 cells were cultivated in an atmosphere with 5% CO_2_. Before treatment with AcSDKP, cells were switched to serum-free DMEM to prevent the breakdown of AcSDKP by ACE, present in the serum.

### Patient Samples

Inflamed and non-inflamed colonic mucosal samples were collected from patients with active UC (n = 13), who underwent colonoscopy at the Department of Gastroenterology, Xinhua Hospital (Shanghai, China) from July 2015 to June 2019. The basic characteristics of the patients are shown in **Table 1**. Additionally, inflamed and paired distant non-inflamed intestinal tissues were collected from UC patients (n = 5) when they received surgery. The collection and usage of the aforementioned human tissues were approved by the Research and Ethics Committee of Xinhua Hospital, and the study was conducted in accordance with the relevant guidelines. All patients provided written informed consent according to the Declaration of Helsinki ahead of the study.

**Table 1:** Basic information of A-UC patients.

	Active-UC (n = 13)
Age (years)	
≤30	6
30–50	4
≥50	3
Gender	
Male	7
Female	6
Disease duration (years)	
≤1	5
1–3	5
≥3	3
Location	
Colon	8
Rectum	3
Ileocecal	2
CRP (mg/L)	17.75 ± 5.74

### Mice and Colitis Induction

PREP-KO (PREP^−/−^) mice, with C57BL/6 background, were generated from Shanghai Model Organisms Center (Shanghai, China). The mice were genotyped by PCR analysis, using the primer sets P1/P2 and P3/P4. The detailed sequences of primer sets are listed in **Table 2**, and the representative PCR products are presented in **Supplementary Figure 1**. The PREP-KO mice resembled the WT mice in phenotype. The female C57BL/6 mice (6–8 weeks old, WT) were purchased from the Experimental Animal Center of the Chinese Academy of Sciences (Shanghai, China) and were allowed to acclimatize for nearly two weeks before the experiment. All the animals were raised in a specific pathogen-free facility and permitted to free diet. Addition of 2.5% or 3% dextran sulfate sodium (DSS) (36–50 kDa) to the drinking water induced experimental colitis in mice. Every mouse was assessed daily based on the disease activity index (DAI), which included weight loss, stool consistency, and fecal blood. The scoring system for calculating DAI and histological score are shown in **Table 3** and **Table 4**, respectively, as described previously (Wirtz et al., 2017). Mice were finally sacrificed by CO_2_ suffocation. All animal experiments were approved by the Institutional Animal Care and Use Committee of Xinhua Hospital and carried out in accordance with the relevant guidelines.

**Table 2:** The sequences of primers used in this study.

Primers	Forward (5′-3′)	Reverse (5′-3′)
PREP-MICE-P1/P2	TACCGCTACCCCTGCTTCA	GCTATGTCGGCTCCAACCA
PREP-MICE-P3/P4	AGCTACTTCCTGCCCCCTCTTAC	GGAATCCCCAACACTGACACAAA
TNF-α (mice)	CAGGCGGTGCCTATGTCTC	CGATCACCCCGAAGTTCAGTAG
IL-1β (mice)	GCAACTGTTCCTGAACTCAACT	ATCTTTTGGGGTCCGTCAACT
IL-6 (mice)	TAGTCCTTCCTACCCCAATTTCC	TTGGTCCTTAGCCACTCCTTC
ACE (mice)	AGAGTACAACCAGATCCTGCTAGAC	TCCAGCTCTTCCATGCCCATAG
ACE (human)	GACGATCTGGAACACCTCTAC	AGCATAGTACTGGTGACATCG
MCP-1 (human)	CAAGCAGAAGTGGGTTCAGGATT	TCTTGGGTTGTGGAGTGAGTGTTC
IL-8 (human)	CTGCGCCAACACAGAAATTA	TGAATTCTCAGCCCTCTTCAA
TNF-α (human)	CCTCTCTCTAATCAGCCCTCTG	GAGGACCTGGGAGTAGATGAG
IL-6 (human)	ACTCACCTCTTCAGAACGAATTG	CCATCTTTGGAAGGTTCAGGTTG
Tβ4 (human)	ATGTCTGACAAACCCGATATGGC	CCAGCTTGCTTCTCTTGTTCA
PREP (human)	CATCTCCCAAGAGGCTGACTA	GGGCAATAACACAACCAAAGA
Meprin-α (human)	ATTTCAACAGTTTGATGGGTGCT	ATGGCCTTATAGGCACATCCT
GAPDH (mice)	AGGTCGGTGTGAACGGATTTG	GGGGTCGTTGATGGCAACA
GAPDH (human)	GGAGCGAGATCCCTCCAAAAT	GGCTGTTGTCATACTTCTCATG

**Table 3:** Detailed scoring system for assessing the disease activity index (DAI).

Score	Body weight loss	Stool	Bleeding
0	None	Normal	No bleeding
1	1–5%	Soft but still formed	No bleeding
2	6–10%	Soft	Positive hemoccult
3	11–18%	Very soft or wet	Visible blood traces
4	> 18%	Watery diarrhea	Gross bleeding

DAI scores = body weight loss scores+ stool scores + bleeding scores.

**Table 4:** Detailed scoring system for assessing the histological changes.

Score	Tissue damage (T)	Inflammatory cell infiltration (I)
0	None	None
1	Isolated focal damage	Increased
2	Mucosal erosions and ulcerations	Infiltration of the submucosa
3	Extensive damage deep into the wall	Transmural infiltrations

Histological score = T+ I.

### AcSDKP Delivery

Female wild-type C57BL/6 mice (aged 8–10 weeks) were randomly divided into five groups: (1) control, (2) control + AcSDKP, (3) DSS, (4) DSS + vehicle, (5) DSS + AcSDKP. Based on prior studies (Worou et al., 2015) and our preliminary experiment, AcSDKP infusion was administered intraperitoneally through mini-osmotic pumps (Alzet, model 1002, Cupertino, CA), at a dose of 1,600 μg/kg/day. Two days before colitis induction, mice in group 2 and group 5 were anesthetized with isoflurane. Subsequently, mini-osmotic pumps containing AcSDKP were surgically implanted in their abdominal cavity, under aseptic conditions. The mice in group 4 underwent a similar surgical procedure, except that the pump was filled with saline.

### Western Blots

Proteins (20–40 μg/well) were loaded onto 8% to 12% SDS-PAGE for electrophoresis and then transferred to PVDF membranes (Millipore, USA). Next, the membranes were blocked and incubated overnight with specific primary antibody at 4°C. After washing with tris borate saline plus 0.1% tween 20 (TBST), the membranes were incubated with corresponding secondary antibody for 80 min and again washed with TBST. Finally, the signals were detected with ECL chemiluminescence reagents (Bio-Rad, USA) by a ChemiDoc™ XRS^+^ System (Bio-Rad, USA). Levels of target proteins were normalized to β-actin and analyzed by Image Lab software. The primary antibodies used in the study are shown in **Supplementary Table 1**.

### Quantitative Real-Time PCR

Total RNA was extracted using Trizol (Invitrogen, USA), and quantified according to the manufacturer's instructions. cDNA was synthesized from 1 μg of RNA, using the PrimeScript™ RT Master Mix (Takara, Japan). qRT-PCR was performed utilizing a TB Green Premix Ex Taq™ (Takara, Japan) on the PikoReal RT-PCR System (Thermo, USA). Data were analyzed by the 2^−ΔΔCt^ method. The detailed sequences of primers are provided in **Table 2**.

### Immunohistochemistry

Tissue sections were first deparaffinized in dimethylbenzene and rehydrated in different concentrations of ethanol. Next, heat-induced antigen retrieval was carried out in citrate buffer, followed by quenching of endogenous peroxidases. After blocking non-specific binding with 3% BSA, the sections were incubated overnight with primary antibody (**Supplementary Table 1**) at 4°C. Subsequently, the sections were incubated with HRP-conjugated secondary antibody for 50 min, and immunoreactivity was visualized with 3,3′-diaminobenzidine. Finally, the nuclei were counterstained, and the sections were dehydrated in ethanol series. Images of all the sections were captured under the same microscope (Olympus, Japan).

### Determination of Colonic AcSDKP Levels

AcSDKP levels in the full-thickness colon were detected with a highly specific enzyme immunoassay (EIA) kit (SPI-bio, France). Colon tissues were homogenized in RIPA lysis buffer, containing 1 mmol/L phenylmethanesulfonyl fluoride and 10^−5^ mol/L captopril. The lysates was centrifuged at 14,000 rpm for 15 min to obtain the supernatant. Total protein concentration was quantified by the BCA Protein Assay Kit (Takara, Japan). Then, 0.15 ml of the supernatant was extracted with 1 ml of methanol and dried by vacuum centrifugation. After reconstitution with EIA buffer, the AcSDKP levels in the sample were determined as per the manufacturer's protocol and expressed as nmol/g colon protein.

### ACE Activity Assay

ACE activity in the colon of mice was measured using an ACE activity assay kit (Sigma, USA). Briefly, tissue samples were prepared using an appropriate lysis buffer, and total protein concentration was determined. Next, samples and positive controls were added to the wells, and the standard curve was conducted. The reaction was initiated by adding the diluted substrate to the sample wells. The fluorescence was measured immediately in a kinetic mode. Values were expressed in U/mg protein.

### Statistical Analysis

All experiments were performed in triplicate. Data are shown as mean ± standard error of the mean (SEM). Comparisons between two or more groups were achieved using Student's *t*-test, one-way analysis of variance (ANOVA) or Mann-Whitney U test. SPSS 23.0 software was used to carry out all statistical analyses. P values < 0.05 were considered statistically significant.

## Results

### Tβ4 and Meprin-α Expression Are Decreased in Inflamed Intestinal Tissues From UC Patients

**Figure 1:**
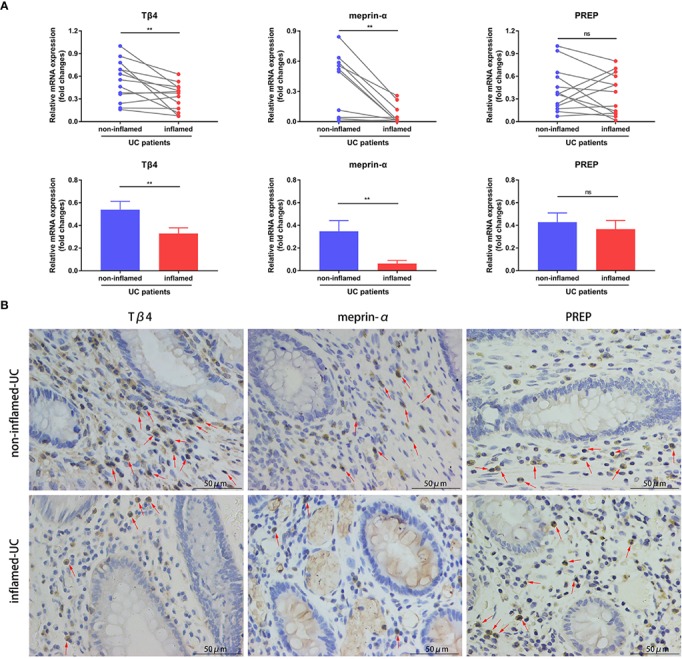
Expression of Tβ4, meprin-α, and PREP in the intestinal tissues of UC patients. **(A)** Quantitative RT-PCR analysis of Tβ4, meprin-α and PREP in the inflamed and non-inflamed colonic mucosa from the same patients with A-UC (n = 11–13). Data are expressed as mean ± SEM, and statistical analysis was performed by paired t-test. **P < 0.01, ns, not significant. **(B)** Representative immunohistochemical images of Tβ4, meprin-α, and PREP in the paired inflamed and non-inflamed intestinal tissues of UC patients (n = 5). Red arrows indicate positive expression. Scale bars correspond to 50 μm. All data are shown from three independent experiments.

To determine whether colonic AcSDKP level correlates with inflammation in IBD patients, we first examined the expression of Tβ4, the precursor of AcSDKP, in paired inflamed and non-inflamed biopsy mucosa from A-UC patients. As shown in **Figure 1A**, the mRNA levels of Tβ4 were lower in the inflamed group compared with the non-inflamed group. Next, we measured the expression of meprin-α and PREP, which are the main enzymes responsible for hydrolyzing Tβ4 into AcSDKP. We found that meprin-α mRNA expression was markedly decreased in the inflamed mucosa of A-UC patients compared with the non-inflamed mucosa from the same patients (**Figure 1A**). In contrast, the expression of PREP was similar in the inflamed and non-inflamed mucosa of patients with A-UC (**Figure 1A**). These observations were further verified by immunohistochemistry in five pairs of inflamed and non-inflamed intestinal tissues from UC patients (**Figure 1B**). Moreover, our results showed that ACE mRNA expression was also similar in the inflamed and non-inflamed mucosa from A-UC patients (**Supplementary Figure 2**). Collectively, our findings suggest that AcSDKP concentration may be reduced in the inflamed intestinal tissues of UC patients, owing to the lower rate of generation.

### AcSDKP Inhibits Tumor Necrosis Factor-α–Stimulated Inflammatory Responses of Caco-2 Cells and Represses the Activation of MEK-ERK Signaling

**Figure 2:**
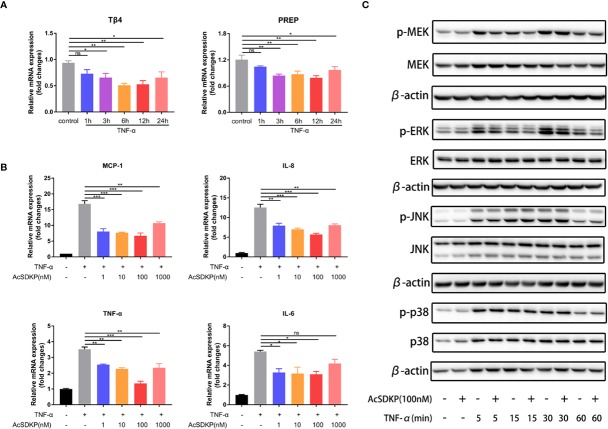
AcSDKP exerts an anti-inflammatory effect on IECs. **(A)** The dynamic changes in the expression of Tβ4 and PREP in Caco-2 cells exposed to TNF-α (10 ng/ml) were determined by qRT-PCR. Data are shown as mean ± SEM from three independent experiments. *P < 0.05, **P < 0.01, ns, not significant versus control group. **(B)** Caco-2 cells were stimulated with TNF-α (10 ng/ml) for 3 h to assess the mRNA expression of inflammatory cytokines and chemokines (MCP-1, IL-8, TNF-α, IL-1β) by qRT-PCR. Data are expressed as mean ± SEM and replicated at least three times. *P < 0.05, **P < 0.01, ***P < 0.001, ns, not significant versus TNF-α group. **(C)** The protein levels of p-p38, p38, p-JNK, JNK, p-ERK, ERK, p-MEK, MEK in Caco-2 cells stimulated with TNF-α (10 ng/ml) were analyzed by western blot at different time points. Representative images from three independent experiments with similar results are shown above. All statistical analyses were performed by one-way ANOVA.

To investigate whether AcSDKP is able to regulate intestinal inflammation *in vitro*, we initially used tumor necrosis factor-α (TNF-α) to stimulate the Caco-2 cells and then examined the expression of Tβ4, meprin-α and PREP in Caco-2 cells under an inflammatory state. The levels of both Tβ4 and PREP were remarkably downregulated as a consequence of TNF-α stimulation, especially after 6 and 12 h (**Figure 2A**). The mRNA expression of meprin-α was negligible in Caco-2 cells, under either normal conditions or inflammatory state (data not shown). These results indicate that AcSDKP content was either low or negligible in IECs. Next, we explored the effect of AcSDKP on the inflammatory responses of Caco-2 cells. As shown in **Figure 2B**, TNF-α administration greatly increased the mRNA expression levels of MCP-1, IL-8, TNF-α, and IL-6 in Caco-2 cells, which was partly reversed by AcSDKP in a concentration-dependent manner. The strongest inhibitory effect of AcSDKP appeared at the dose of 100 nM. Based on our findings and previous studies, we used the dose of 100 nM AcSDKP for the subsequent experiment. Given that the mitogen-activated protein kinase (MAPK) pathway is capable of modulating the gene expression of multiple inflammatory cytokines, we further tested the influence of AcSDKP on the activation of ERK, JNK, and p38, which are the three main subfamilies of MAPK. Western blot analysis revealed that AcSDKP dramatically inhibited the phosphorylation of ERK in TNF-α-primed Caco-2 cells, whereas AcSDKP failed to reduce JNK and p38 phosphorylation (**Figure 2C**). In addition, AcSDKP also downregulated the phosphorylation of MEK, the dominating upstream kinase of ERK, at 5 and 15 min after TNF-α induction (**Figure 2C**). Taken together, these results imply that AcSDKP can attenuate inflammatory responses of IECs *via* inhibition of MEK-ERK signaling.

### Inhibition of AcSDKP Production Renders Mice More Susceptible to DSS-Induced Colitis

**Figure 3:**
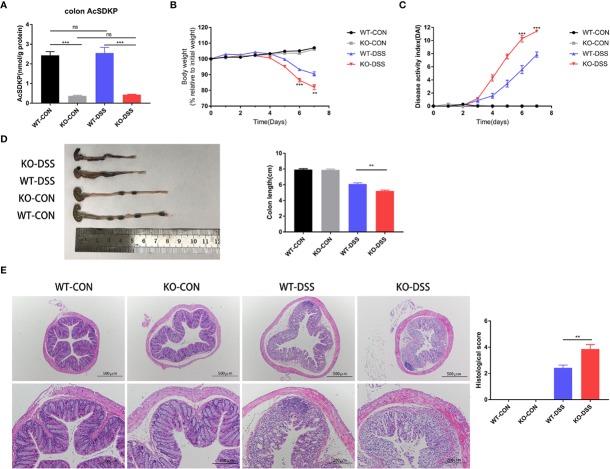
Inhibition of AcSDKP production increases the susceptibility of mice to experimental colitis. PREP-KO and WT male mice (6–8 weeks old, 20–24 g) were treated with normal drinking water or 2.5% DSS-containing water for 7 days and were sacrificed on day 8 (n = 7 for each group). **(A)** The level of AcSDKP in the colon was detected by EIA. **(B)** Body weight loss and **(C)** Disease activity index (DAI) were assessed daily. **(D)** Macroscopic appearance and the length of the colon were examined at day 8. **(E)** Representative hematoxylin and eosin (H&E) staining images of the distal colon and histological scores. Scale bars correspond to 500 and 200 μm. Data are representative of three independent experiments. Statistical analysis between two groups was performed by unpaired t-test or Mann-Whitney U test. **P < 0.01, ***P < 0.001, ns, not significant versus WT-DSS group.

To uncover the potential role of AcSDKP in the pathogenesis of colitis, we knocked out the gene of PREP to inhibit the generation of AcSDKP in mice. As expected, the basal AcSDKP level was significantly lower in the colonic tissues from PREP-KO mice (**Figure 3A**). The homozygous PREP-KO mice exhibited no obvious anatomical or histological abnormalities in the intestine. Of note, no spontaneous colitis was observed in PREP-KO mice. Next, PREP-KO male mice and WT male littermates were exposed to 2.5% DSS for 7 days to induce UC-like colitis. We found that PREP-KO mice started losing weight earlier than WT mice and lost a large percentage of body weight by the end of the experiment (**Figure 3B**). Moreover, PREP-KO mice displayed higher DAI scores and shorter colon lengths than the WT littermates (**Figures 3C, D**). Histological examination of distal colonic tissues from PREP-KO mice also showed more severe pathological changes compared with that in the WT mice, as evidenced by wider disruption of crypt architecture and more inflammatory cell infiltration (**Figure 3E**). However, there was no variation in the concentration of colonic AcSDKP before and after DSS treatment, in either PREP-KO mice or WT mice (**Figure 3A**). Additionally, both the expression and activity of ACE were similar in the colon of PREP-KO mice and WT mice (**Supplementary Figures 3A, B**). Overall, these data indicate that PREP deficiency accelerates the development of DSS-induced colitis in mice, which is likely attributed to the decreased level of AcSDKP.

### Inhibition of AcSDKP Production Aggravates Intestinal Inflammation in DSS-Induced Mice

**Figure 4:**
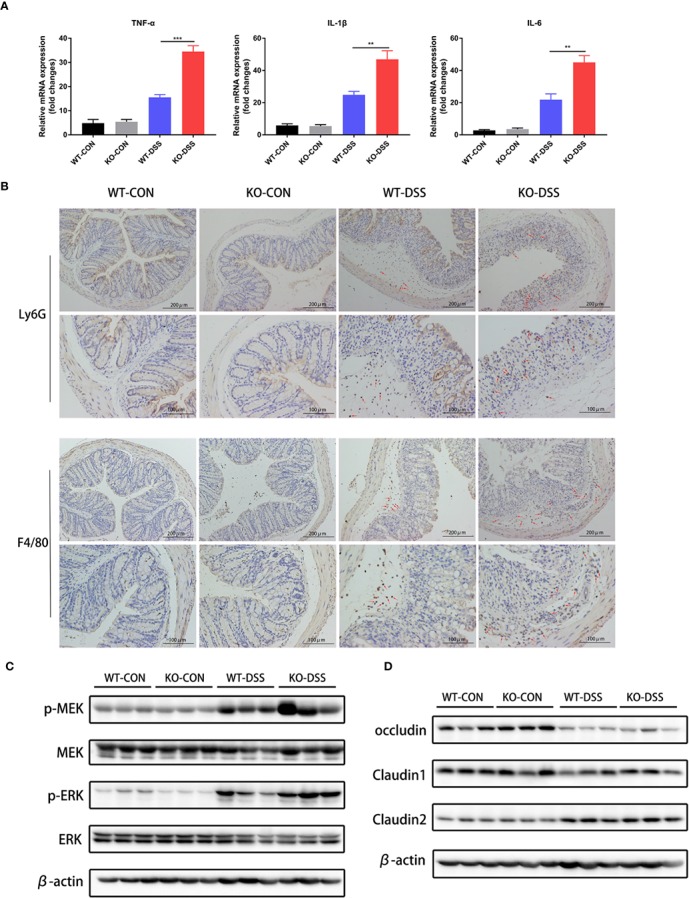
Inhibition of AcSDKP production aggravates intestinal inflammation during experimental colitis. The experimental design is the same as in **Figure 3**. **(A)** Relative mRNA expression of pro-inflammatory cytokines (TNF-α, IL-1β, IL-6) was determined in the colon of PREP-KO and WT mice with or without DSS-exposure using qRT-PCR analysis (n = 7 per group). Data are shown as mean ± SEM and statistical analysis between two single groups was performed by unpaired t-test. **P < 0.01, ***P < 0.001. **(B)** Immunohistochemical analysis of Ly6G^+^ neutrophils and F4/80^+^ macrophages in colonic tissues of mice. Red arrows indicate positive expression. Scale bars correspond to 200 and 100 μm. **(C)** The protein levels of p-MEK, MEK, p-ERK, and ERK were determined using western blot. **(D)** Western blot analysis for occludin, claudin1, and claudin2 in the colonic tissues. All data are representative of three independent experiments.

Seeing that the uncontrolled release of proinflammatory cytokines and excessive accumulation of inflammatory cells are important factors in intestinal inflammation of IBD, we further assessed the mRNA expression levels of TNF-α, IL-1β, and IL-6 in the colon of PREP-KO mice and WT mice, as well as the degree of neutrophils and macrophages infiltration. As shown in **Figure 4A**, DSS treatment significantly induced the expression of proinflammatory cytokines (TNF-α, IL-1β, IL-6) in the colon of both PREP-KO mice and WT mice, but obviously PREP-KO mice exhibited a greater elevation of these cytokines. In addition, a larger number of infiltrating Ly6G^+^ neutrophils and F4/80^+^ macrophages accumulated in the colon of PREP-KO mice compared to that in WT mice (**Figure 4B**). Consistent with previous findings in Caco-2 cells, the levels of p-ERK and p-MEK were prominently higher in the colon of PREP-KO mice than in WT mice (**Figure 4C**). Furthermore, increased expression of collagen I and collagen III was detected in the colon of PREP-KO mice compared with that in WT mice (**Supplementary Figure 3C**). Taking into consideration the integral connection of impaired intestinal epithelial barrier with the sustained intestinal inflammation, we speculated whether the decline in colonic AcSDKP level had any effects on the protein level of tight junction proteins in colonic tissues. However, there were no evident differences in the protein levels of occludin, claudin1, or claudin2 between PREP-KO mice and WT mice (**Figure 4D**). Overall, these results suggest that inhibition of AcSDKP production exacerbates the DSS-induced colonic inflammation in mice.

### Exogenous AcSDKP Infusion Alleviates Experimental Colitis in Mice Induced by DSS

**Figure 5:**
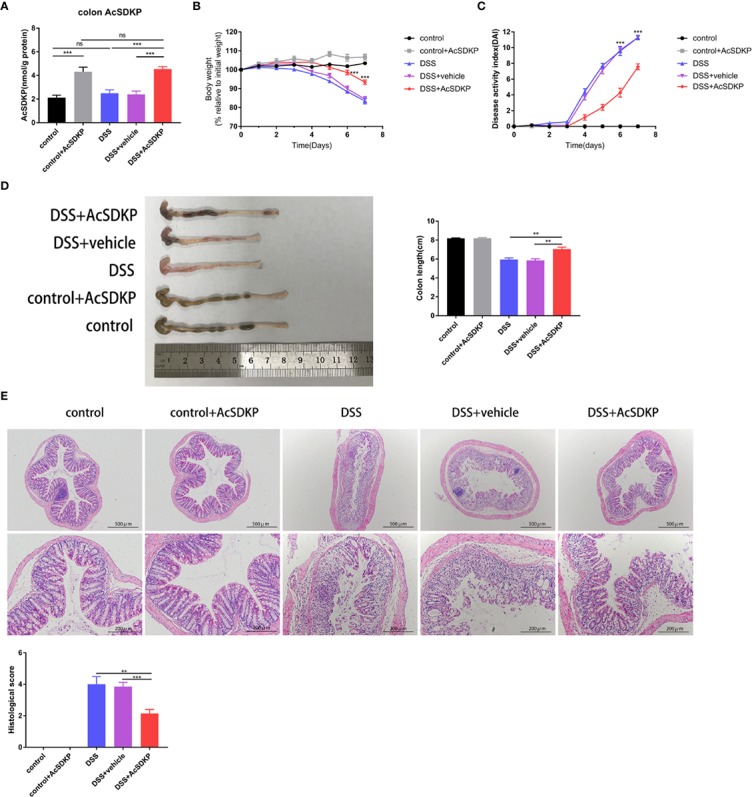
AcSDKP ameliorates DSS-induced acute colitis in mice. The female C57BL/6 mice (8–10 weeks old, 18–22 g) received AcSDKP-infusion starting 2 days before DSS administration and continuing until the end of experiments (n = 5 for AcSDKP group, n = 7 for other groups). **(A)** The colonic AcSDKP level was monitored by EIA. **(B)** Body weight change and **(C)** DAI scores were recorded on a daily basis. **P < 0.01, ***P < 0.001 versus DSS + vehicle group. **(D)** Gross morphology images of the colon from mice at the end of modeling and colon length were measured. **(E)** The distal colon sections were stained with H&E, and histological scores were assessed accordingly. Scale bars correspond to 500 and 200 μm. Data are representative of three independent experiments. Statistical analysis between groups was performed by unpaired t-test, one-way ANOVA or Mann-Whitney U test.

To further ascertain whether AcSDKP plays an anti-inflammatory role in the intestine, which accounts for the increased vulnerability of PREP-KO mice to experimental colitis, female WT mice were treated with AcSDKP (1,600 μg/kg/day) or vehicle (saline solution) *via* mini-osmotic pumps, followed by 3% DSS induction. As confirmed in **Figure 5A**, the colonic AcSDKP content was almost two-fold higher in mice that received a continuous infusion of exogenous AcSDKP compared with those that did not receive AcSDKP, in both DSS-treated group and control group. We found that AcSDKP significantly attenuated weight loss and colon shrinking resulted from DSS (**Figures 5B, D**). Moreover, the DAI score was decreased upon AcSDKP administrating (**Figure 5C**). Consistently, AcSDKP-treated mice showed lower histopathological scores in terms of tissue damage and inflammatory cell infiltration (**Figure 5E**). Besides, we found that AcSDKP treatment did not affect the expression and activity of ACE in the colonic tissues from mice (**Supplementary Figures 4A, B**). Taken together, these results imply that enhancing the colonic AcSDKP concentration helped in reducing the severity of DSS-induced colitis.

### Exogenous AcSDKP Infusion Relieves Inflammatory Responses in the Colon

**Figure 6:**
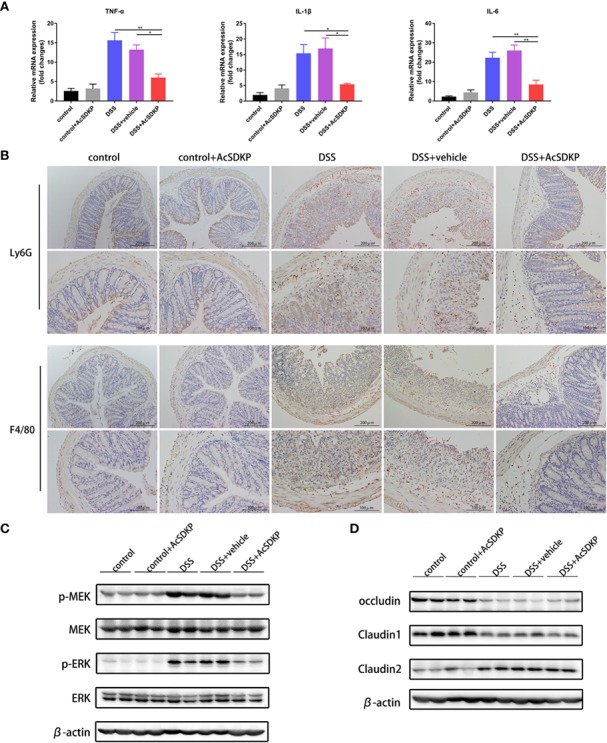
AcSDKP restricts inflammatory responses in the colon caused by DSS. The experimental design is the same as in **Figure 5**. **(A)** Relative mRNA levels of pro-inflammatory cytokines (TNF-α, IL-1β, IL-6) were determined in the colon *via* qRT-PCR (n = 5 per group). Statistical analysis was performed by one-way ANOVA. *P < 0.05, **P < 0.01 versus DSS group or DSS + vehicle group. **(B)** Immunohistochemistry staining of Ly6G^+^ neutrophils and F4/80^+^ macrophages in paraffin-embedded colon sections. Red arrows indicate positive expression. Scale bars correspond to 200 and 100 μm. **(C)** Western blot analysis for p-MEK, MEK, p-ERK, and ERK in the colonic tissues. **(D)** The protein levels of occludin, claudin1, and claudin2 were determined using western blot analyses. Data are representative of three independent experiments.

Similar to previous studies in PREP-KO mice, we explored the effects of increased AcSDKP levels on the intestinal immune environment. As shown in **Figure 6A**, AcSDKP treatment led to a profound decrease in the levels of TNF-α, IL-1β, and IL-6. The colon of AcSDKP-treated mice consistently had fewer Ly6G^+^ neutrophils and F4/80^+^ macrophages (**Figure 6B**). In agreement with the previous *in vitro* data, AcSDKP also inhibited the phosphorylation of ERK and MEK in the DSS-triggered colon inflammation (**Figure 6C**). Moreover, AcSDKP-treated mice exhibited reduced expression of collagen I and collagen III in the colon compared with saline-treated mice (**Supplementary Figure 4C**). In addition, we reinvestigated whether AcSDKP modifies the protein level of tight junction proteins in the colonic tissues. However, we failed to detect any significant differences in the expression of occludin, claudin1, or claudin2 upon AcSDKP treatment (**Figure 6D**). Collectively, these data suggest that AcSDKP mitigates symptoms of DSS-induced colitis directly by restricting inflammatory responses, rather than improving the function of the intestinal epithelial barrier.

## Discussion

AcSDKP, a naturally occurring tetrapeptide possessing anti-inflammatory and immunomodulatory properties, has shown potential to become a new biomarker of inflammation (Ntsekhe et al., 2012). In this study, we demonstrated that AcSDKP suppressed TNF-α-induced inflammatory responses of IECs partly by inhibiting the activation of MEK-ERK signaling. More importantly, our results proved that AcSDKP played a protective role in experimental colitis in mice. In addition, our data raised the possibility that AcSDKP content may be downregulated in the inflamed colonic tissues of IBD patients.

In tissues, the concentration of AcSDKP is maintained by a balance between its generation and degradation. Tβ4, an endogenous 43-amino acid peptide known as G-actin sequestering protein, is considered as the major source of AcSDKP (Liu et al., 2010; Kumar et al., 2018). Our results showed that Tβ4 levels were lower in the inflamed mucosa of A-UC patients compared with that in the non-inflamed mucosa. Although the detailed mechanisms remain unclear, two main enzymes participating in the production of AcSDKP from Tβ4 have been identified: meprin-α and PREP (Kumar et al., 2016). Herein, we discovered that there was no obvious difference in the expression level of PREP between inflamed and non-inflamed tissues of A-UC patients. These results are in line with a prior study that found that PREP activity in the colonic tissues of healthy controls was similar to those in the affected and non-affected tissues of IBD patients (Koelink et al., 2014). *MEP1A*, a gene that encodes the α subunit of meprin-α, has been reported as a downregulated gene in human IBD and a decreased level of meprin-α is related to augmented intestinal inflammation in IBD patients (Banerjee et al., 2009; Clark et al., 2012). In our study too, we found that lower levels of meprin-α were associated with increased inflammation of the intestine. On the other hand, ACE is the main enzyme responsible for the degradation of AcSDKP. Previous studies found that serum ACE activity tended to be higher in A-UC patients who did not receive steroidal therapy, compared with healthy controls or patients with inactive UC (Silverstein et al., 1981); and there were no significant differences in circulating ACE activity or mucosal ACE expression between UC (including active and inactive) or CD patients and healthy controls (Silverstein et al., 1981; Garg et al., 2015; Garg et al., 2020). Among A-UC patients, we detected that the mRNA level of ACE was similar in the inflamed and non-inflamed mucosa. Overall, these data suggest that there might be a reduction in the AcSDKP content in the inflamed colonic region of IBD patients.

It is widely established that the dysfunction of IECs plays a critical role in either the etiology or the pathology of IBD, in part by producing various inflammatory cytokines which mediate the cross-talk between IECs and immune cells (Peterson and Artis, 2014). Herein, we observed that AcSDKP inhibited TNF-α-induced inflammatory cytokines and chemokines expression at mRNA levels in Caco-2 colonic adenoma cells, and the inhibitory effect was dependent on the concentration of AcSDKP. To further elucidate the underlying molecular mechanisms, we explored the impact of AcSDKP on the important subfamilies of MAPK, which can be activated by diverse stimuli, including cytokines of TNF family and then regulate the production of multiple cytokines (Kyriakis and Avruch, 2012). Intriguingly, we discovered that AcSDKP repressed the phosphorylation of ERK and MEK in Caco-2 cells exposed to TNF-α, but did not influence the phosphorylation of JNK and p38. Combined with the inhibitory effects of ERK inhibitor on the expression and secretion of IL-8 in Caco-2 cells (Sunil et al., 2010), our results suggest that the anti-inflammatory effect of AcSDKP is probably through inhibition of MEK-ERK signaling and is independent of JNK and p38.

DSS-induced colitis model is a widely used animal model for the investigation of pathogenesis and therapeutics of IBD. In this study, two complementary methods were used to study the exact role of AcSDKP in DSS-induced colitis. We found that basal AcSDKP content was dramatically lower in the colon of PREP-KO mice compared with WT mice, indicating that PREP is indispensable for the generation of AcSDKP under physiological conditions. PREP deficient mice showed enhanced susceptibility to DSS-induced colitis and displayed more severe colon damage and more robust inflammatory reactions, suggesting that the decreased level of AcSDKP might partly explain the detrimental effects of PREP deficiency. Furthermore, we demonstrated that in WT mice, exogenous AcSDKP infusion ameliorated the clinical symptoms of colitis caused by DSS, accompanied by improved intestinal immune environments, suggesting that AcSDKP indeed exerts an inhibitory effect on intestinal inflammation. Similar to the findings *in vitro*, AcSDKP was capable of blocking the activation of MEK-ERK signaling in DSS-induced mice. A recent study showed that the ERK inhibitor, U0126, significantly blunted the expression of proinflammatory factors and improved the composition of intestinal flora, thereby protecting mice against DSS-induced colitis (Song et al., 2019). Thus, the anti-inflammatory effects of AcSDKP in the intestine may be partly due to its inhibition of MEK-ERK signaling pathway.

In the colon of PREP-KO mice, basal AcSDKP content was not completely absent, albeit at a very low level. This indicated that there may be other peptidases involved in AcSDKP generation cascades, playing a similar but weaker role as PREP. Alternatively, AcSDKP can be formed from peptides other than Tβ4 without the aid of PREP. Considering that basal AcSDKP content in the kidney of mice was almost reduced by half, resulting from meprin-α deficiency, it seemed that PREP contributed more to the production of AcSDKP since PREP deficiency led to a more than 80% reduction of basal AcSDKP level in the colon. Of note, the AcSDKP level in the colon was not significantly affected due to DSS-induced colitis, in either PREP-KO mice or WT mice. This is not unexpected for the reason that the experimental colitis model only mimics the acute episode period of human IBDs (Wirtz et al., 2017).

Undoubtedly, there are several limitations in our present study. First, we analyzed only the precursor and enzymes engaged in the production and breakdown of AcSDKP in human IBD, while measurement of the definite levels of AcSDKP is warranted to accurately elucidate the correlation of AcSDKP level with colitis severity in IBD patients. Second, we cannot rule out the possibility that AcSDKP also modulates other signaling pathways that regulate the expression of inflammatory mediators apart from MEK-ERK signaling. Thus, additional studies are warranted to fully understand the underlying mechanisms explaining the anti-inflammatory effects of AcSDKP. Third, we only explored the effects of AcSDKP on acute intestinal inflammation, and it makes sense to further investigate the functions of AcSDKP in chronic intestinal inflammation.

In summary, our study provides the first evidence that AcSDKP showed strong protective effects in animal models of acute colitis through inhibition of intestinal inflammatory responses. As an endogenous small peptide, AcSDKP is devoid of toxicity not only in experimental animals but also in human patients (Carde et al., 1992). Keeping in view that an analog of AcSDKP that is resistant to ACE degradation has shown similar functions compared with AcSDKP (Ma et al., 2014), we suggest that both AcSDKP and its analogs have a promising perspective in the treatment of IBD, as well as other inflammatory diseases.

## Data Availability Statement

All datasets generated for this study are included in the article/**Supplementary Material**.

## Ethics Statement

The studies involving human participants were reviewed and approved by the Research and Ethics Committee of Xinhua Hospital. The patients/participants provided their written informed consent to participate in this study. The animal study was reviewed and approved by the Institutional Animal Care and Use Committee of Xinhua Hospital.

## Author Contributions

YS conceived the research, performed all experiments, analyzed the data and drafted the manuscript. MZ collected endoscopy biopsy samples and gave some assistance in animal experiments. JY, ZG, JW, and YuC helped to interpret the results of experiments and gave guidance for the experiments. YiC supervised the whole work and revised the manuscript.

## Funding

This work was supported by Xinhua Hospital (17YJJ21).

## Conflict of Interest

The authors declare that the research was conducted in the absence of any commercial or financial relationships that could be construed as a potential conflict of interest.
