# Supplementary material for: N-Acetyl-Seryl-Aspartyl-Lysyl-Proline Mitigates Experimental Colitis Through Inhibition of Intestinal Mucosal Inflammatory Responses via MEK-ERK Signaling
Source: Front Pharmacol. 2020 May 6;11:593. doi: 10.3389/fphar.2020.00593 (PMC7218092; doi:10.3389/fphar.2020.00593)
Supplement: Supplementary file 1 [file DataSheet_1.docx]

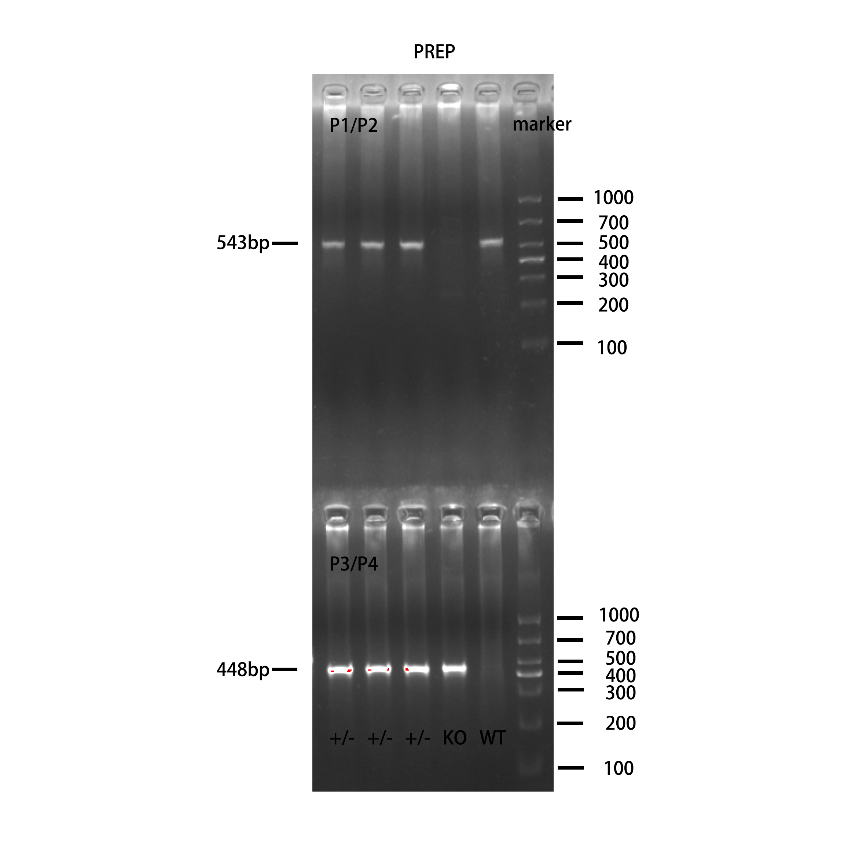


**Supplementary Figure 1.** The genotype of mice was analyzed by PCR.

**
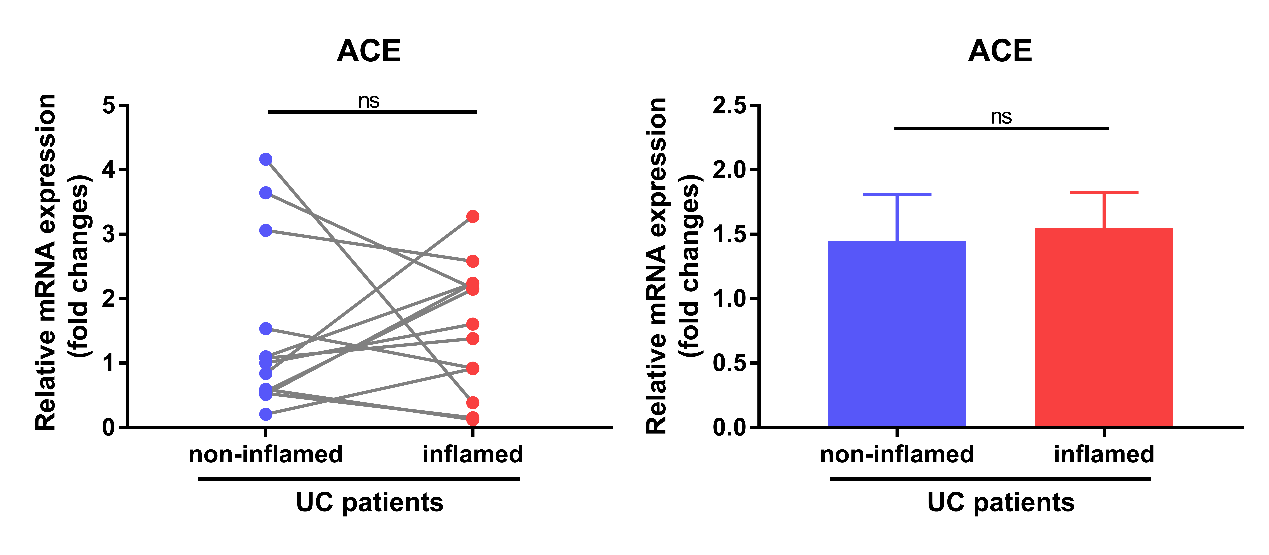
**

**Supplementary Figure 2.** Expression of ACE in the intestinal tissues of UC patients. The mRNA level of ACE was analyzed by quantitative RT-PCR in the inflamed and non-inflamed colonic mucosa from A-UC patients (n=13). Data are expressed as mean ± SEM and statistical analysis was performed by paired t-test. ns = not significant.


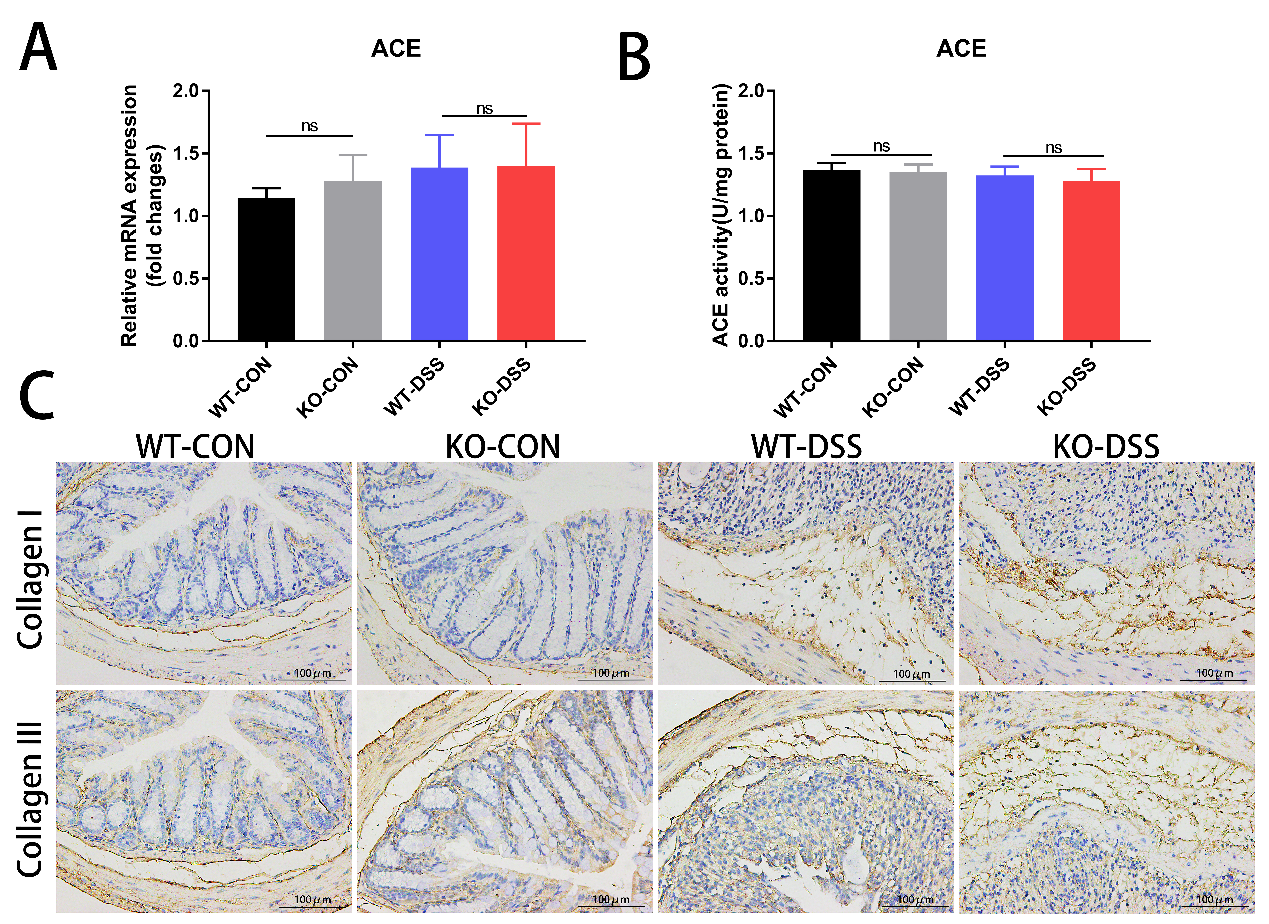


**Supplementary Figure 3.** Inhibition of AcSDKP production increased the colonic expression of collagen I and collagen III in DSS-induced mice, without affecting the expression and activity of ACE. **(A)** Quantitative RT-PCR analysis was used to assess the expression of ACE (n=7 per group). Data are shown as mean ± SEM and statistical analysis between two single groups was performed by unpaired t-test. ns = not significant. **(B)** The activity of ACE was determined using ACE assay kit. 1 U corresponds to 1 nmol/min. **(C)** Immunohistochemical staining for collagen I and collagen III. Scale bars correspond to 100 μm.


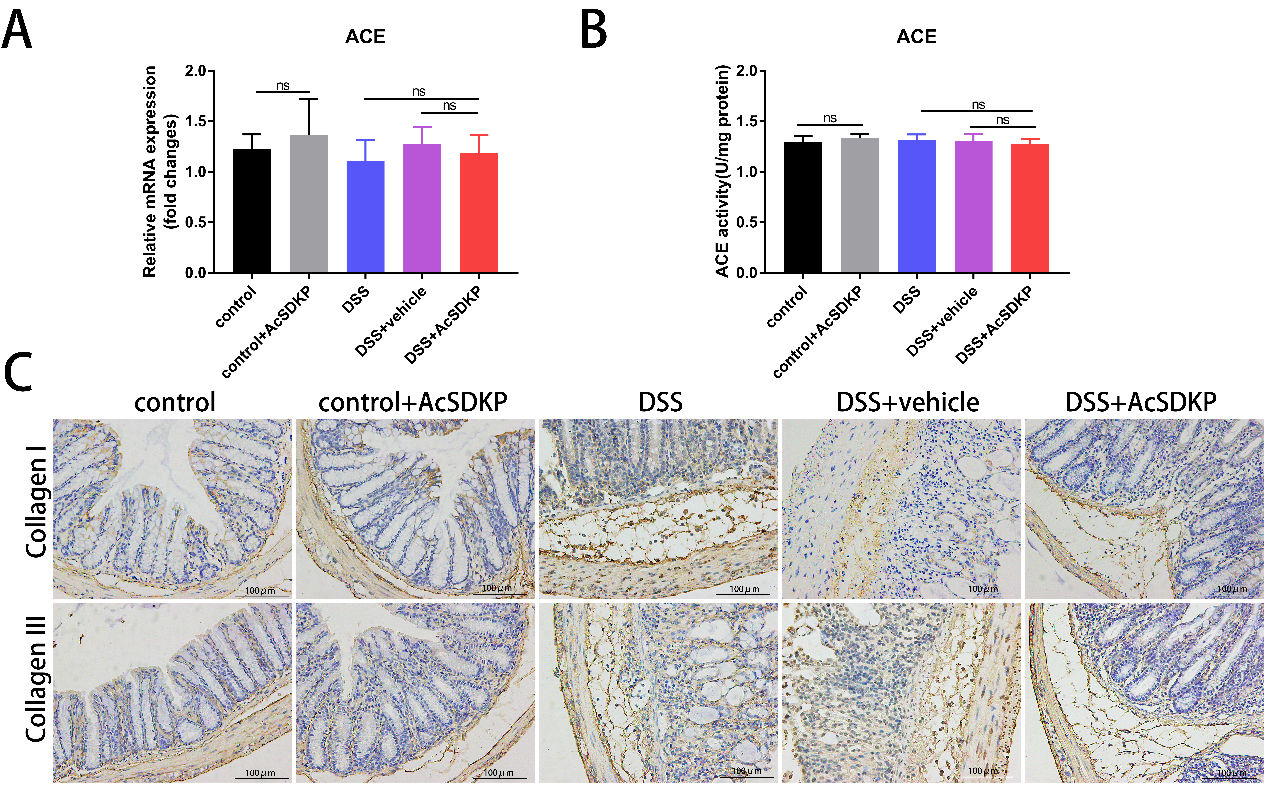


**Supplementary Figure 4.** AcSDKP infusion reduced the colonic expression of collagen I and collagen III in DSS-induced WT mice, without affecting the expression and activity of ACE. **(A)** ACE mRNA expression was determined by quantitative RT-PCR (n=5-7 per group). Data are shown as mean ± SEM and statistical analysis was performed by one-way ANOVA. ns = not significant. **(B)** The activity of ACE was measured using an ACE assay kit. 1 U corresponds to 1 nmol/min. **(C)** Representative immunohistochemical images of collagen I and collagen III. Scale bars correspond to 100 μm.

**Supplementary Table 1.** The antibodies and chemical reagents used in this study.

| Reagents and antibodies | Source | Identifier |
| --- | --- | --- |
| p-MEK (Ser217/221) | Cell Signaling Technology | #9154 |
| MEK | Cell Signaling Technology | #4694 |
| p-ERK (Thr202/Tyr204) | Cell Signaling Technology | #4370 |
| ERK | Cell Signaling Technology | #4695 |
| p-JNK (Thr183/Tyr185) | Cell Signaling Technology | #4668 |
| JNK | Cell Signaling Technology | #9252 |
| p-p38 (Thr180/Tyr182) | Cell Signaling Technology | #4511 |
| p38 | Cell Signaling Technology | #8690 |
| Meprin-α | R&D Systems | AF3220 |
| PREP | Proteintech | 11536-1-AP |
| Tβ4 | Proteintech | 19850-1-AP |
| Occludin | Abcam | ab167161 |
| Claudin1 | Abcam | ab15098 |
| Claudin2 | Thermo Fisher Scientific | 51-6100 |
| β-actin | Cell Signaling Technology | #4970 |
| Ly6G | Servicebio | GB11229 |
| F4/80 | Servicebio | GB11027 |
| Collagen I | Servicebio | GB11022-3 |
| Collagen III | Servicebio | GB13023 |
| DSS | MP Biomedicals | 160110 |
| TNF-α | R&D Systems | 510-RT-010 |
| AcSDKP | Bachem | H-1156.0005 |
| Captopril | Sigma-Aldrich | C4042 |
| AcSDKP EIA kit | SPI-bio | #A05881 |
